# Supplementary material for: Preparation and Characterization of Cardanol-Based Flame Retardant for Enhancing the Flame Retardancy of Epoxy Adhesives
Source: Polymers (Basel). 2022 Nov 29;14(23):5205. doi: 10.3390/polym14235205 (PMC9741169; doi:10.3390/polym14235205)
Supplement: Supplementary file 1 [file polymers-14-05205-s001.zip › polymers-2051720-supplementary.pdf]

Supplementary Materials

Preparation and Characterization of Cardanol-Based Flame Retardant for Enhancing the Flame Retardancy of Epoxy Adhesives

Won-Ji Lee [1], Sang-Ho Cha [1,*] and Do-Hyun Kim [2,*]

[1] Department of Chemical Engineering, Kyonggi University, 154-42, Gwanggyosan-ro, Yeongtong-gu, Suwon 16227, Republic of Korea
[2] Department of Fire Safety Research, Korea Institute of Civil Engineering and Building Technology, 64, 182Beon-Gil, Mado-Ro, Mado-Myeon, Hwaeong-si 18544, Republic of Korea
* Correspondence: sanghocha@kgu.ac.kr (S.-H.C.); kimdh@kict.re.kr (D.-H.K.)

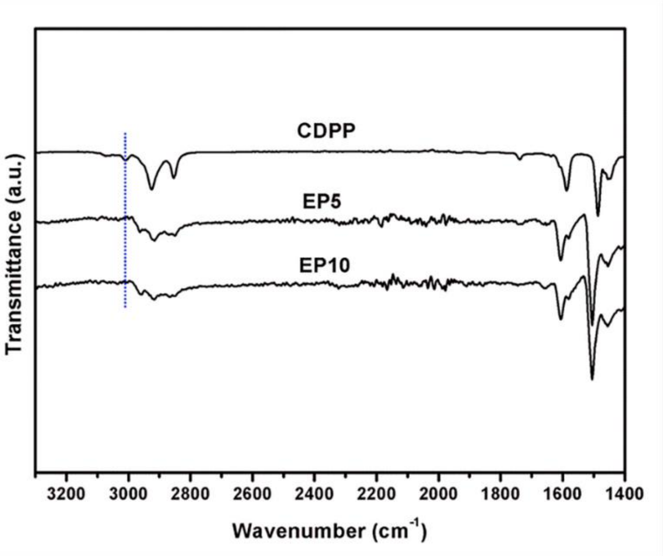

Figure S1. FT-IR spectra of CDPP, EP5, and EP10.

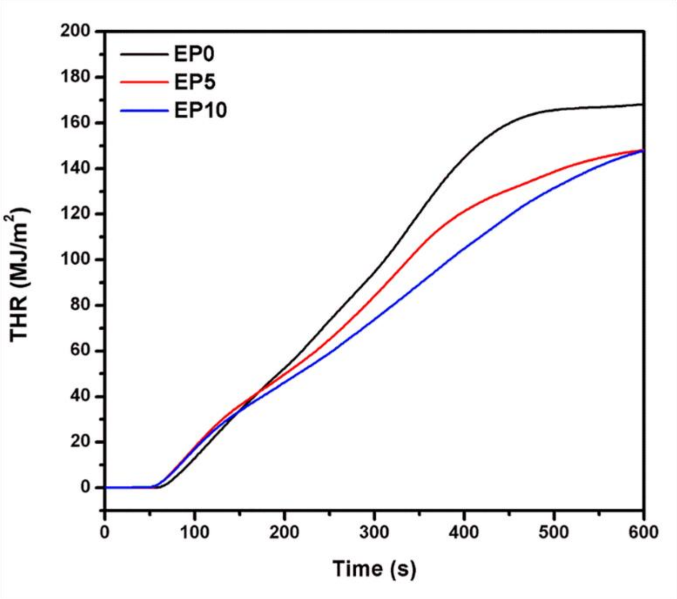

Figure S2. The total heat release (THR) plots of EP series .
